# Under construction: ventral and lateral frontal lobe contributions to value-based decision-making and learning

**DOI:** 10.12688/f1000research.21946.1

**Published:** 2020-02-28

**Authors:** Avinash R Vaidya, Lesley K Fellows

**Affiliations:** 1 Department of Cognitive, Linguistic and Psychological Studies, Brown University, Providence, RI, USA; 2 Montreal Neurological Institute, Department of Neurology & Neurosurgery, McGill University, Montreal, Quebec, Canada

**Keywords:** Neuroeconomics, judgment, decision-making, reward, learning, brain, executive function, neuroimaging, neuropsychology

## Abstract

Even apparently simple choices, like selecting a dessert in a pastry shop, involve options characterized by multiple motivationally relevant attributes. Neuroeconomic research suggests that the human brain may track the subjective value of such options, allowing disparate reward-predictive information to be compared in a common currency. However, the brain mechanisms involved in identifying value-predictive features and combining these to assess the value of each decision option remain unclear. Here, we review recent evidence from studies of multi-attribute decision-making in people with focal frontal lobe damage and in healthy people undergoing functional magnetic resonance imaging. This work suggests that ventromedial and lateral prefrontal cortex and orbitofrontal cortex are important for forming value judgments under conditions of complexity. We discuss studies supporting the involvement of these regions in selecting among and evaluating option attributes during value judgment and decision-making and when learning from reward feedback. These findings are consistent with roles for these regions in guiding value construction. They argue for a more nuanced understanding of how ventral and lateral prefrontal cortex contribute to discovering and recognizing value, processes that are required under the complex conditions typical of many everyday decisions.

## Introduction

**Figure 1.**
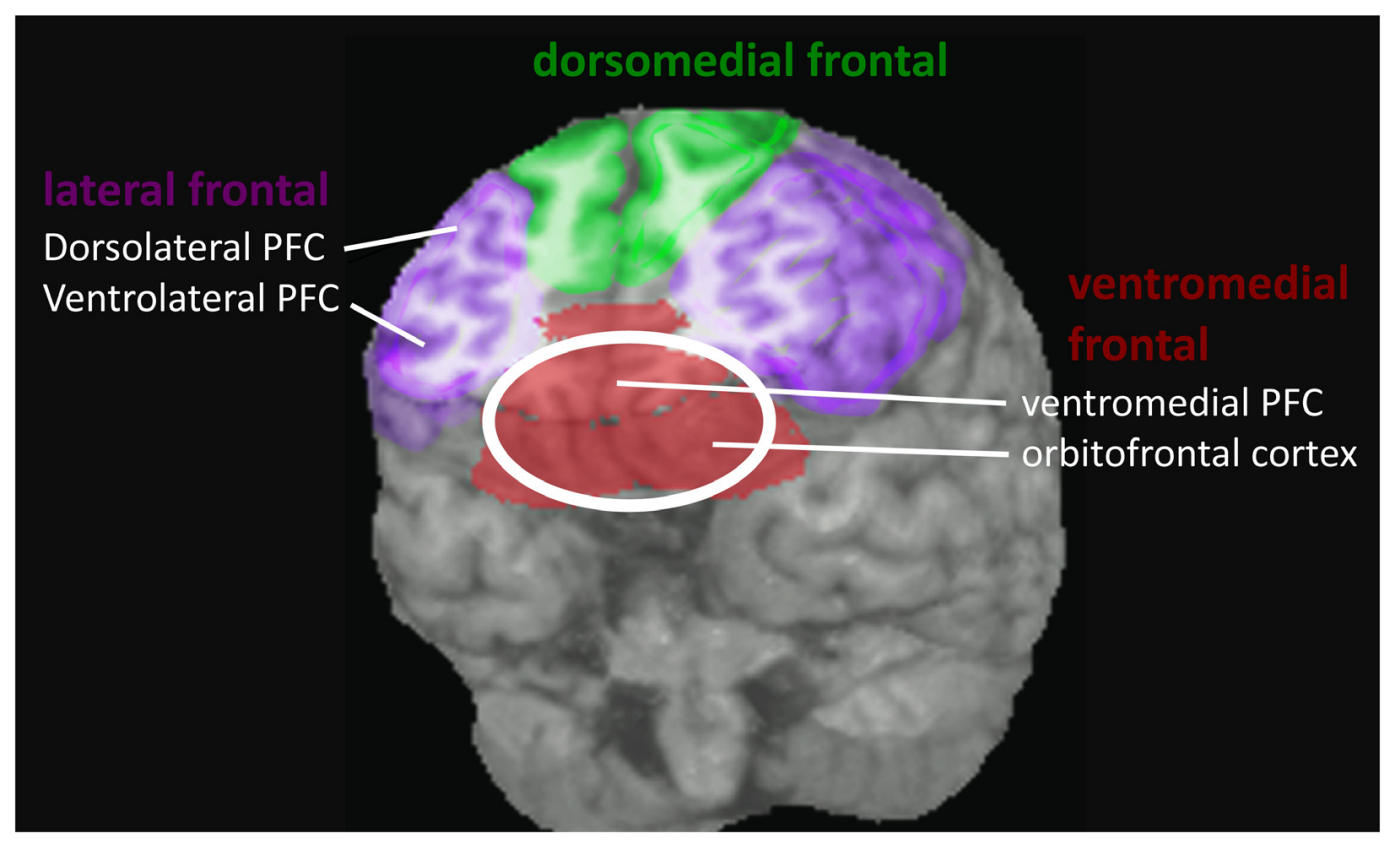
Schematic view of major divisions of the frontal lobes. PFC, prefrontal cortex.

While we easily solve the problem of choosing between cakes lined up in a baker’s display case, it is not clear how our brains make these open-ended choices between complex stimuli. One solution is to find a “value” for each option by integrating various motivationally relevant aspects of each cake into an overall estimate of its subjective rewardingness. These values then can be used to compare all options on a single scale (that is, in a common currency). In the lab, such values typically are quantified in dollars in bidding tasks or as Likert scale ratings. This relatively parsimonious model has been very successful in explaining behavior in many different settings. It has even been applied in algorithms that can learn to perform complex tasks like Atari video games that require maximizing an objective reward (points) ^1^. Certain regions of the frontal lobes—notably the ventromedial prefrontal cortex (vmPFC) and orbitofrontal cortex (OFC)—respond to the values of many different kinds of stimuli ( Figure 1). Such findings have led to the proposal that a common currency value scale might not only be useful for describing behavior but also be implemented as a neural code during decision-making ^2, 3^.

Though elegant, this solution skips over how a buttercream-frosted dessert is converted to a discrete value. Recent work has begun to address the brain processes involved in arriving at subjective value estimates. These studies have examined how the brain notices value-predictive features and learns about and constructs values for the multi-feature objects that are the typical targets of our everyday choices. This line of investigation suggests that values of complex options are not so much carefully calculated as cobbled together or, under some conditions, inferred holistically from a rich slew of perceptual, mnemonic, and hedonic evidence. In this brief review, we will highlight recent functional magnetic resonance imaging (fMRI) and focal lesion studies (in humans) addressing the formation of values for multi-attribute options, whether based on subjective assessment or external goals or learned from reward feedback.

## Reverse-engineering subjective value construction

Much as the history of the Roman Empire can be deciphered from the disparate sources of the limestone and marble that make up its ruins, so too “value” can be deconstructed. Breaking complex options down into their component, value-predictive parts can provide insight into how the brain represents information about the individual elements that contribute to value and how (and where) that information is brought together. This reverse-engineering approach has been applied to diverse decision options: photographs of politicians, familiar and novel foods, artwork, and pseudo-objects.

Functional imaging has found that information about the features or identity of stimuli such as foods, odors, and trinkets is encoded in OFC but that the vmPFC represents a more general value code ^4– 6^. This literature has broadly indicated that vmPFC encodes values independent of stimulus category ^2, 7^. In contrast, OFC value signals appear to be bound more to the lower-level features or attributes of options ^8, 9^. Together, these data are consistent with the notion that vmPFC is involved in integrating information from diverse sources to construct an overall representation of option values.

If vmPFC is critical to developing a common currency value representation from multiple attribute-values, damage to this region should disrupt the ability to combine the values of individual attributes. We observed that patients with damage to the ventromedial frontal lobe (VMF) (subsuming the vmPFC and OFC) made reliable value estimates of complex stimuli but to arrive at those estimates drew on information that was different from that of healthy and frontal lobe–damaged controls. For example, those with VMF damage under-weighted “competence” but relied on “attractiveness” to a similar degree when making decisions about which political candidates to vote for ^10^. Likewise, in a separate study, participants with VMF damage systematically under-weighted some aspects of artwork, such as emotionality, but relied to a similar degree on simpler, visually perceptible attributes such as concreteness and visual balance in comparison with control groups ^11^.

These observations suggest a more nuanced perspective on the role of vmPFC in value judgment. Why do VMF-damaged patients neglect some attribute-values while reliably drawing on others? One possibility is that these attributes depend on latent information built up from conjunctions of simpler, more directly observable features. For example, determining the emotionality of artwork could depend on putting together information about the expressions of the characters with the circumstances in the scene. Both the hippocampus and medial frontal cortex have been implicated in inferring values for novel conjunctions of food ingredients ^12^, implying a role in such a constructive process. We recently found evidence that VMF damage specifically disrupts the ability to choose between multi-attribute pseudo-objects (“fribbles” ^13^) when their values were predicted by the conjunction of two attributes. In contrast, such patients were able to make correct choices when values could be “summed up” on the basis of the values of individual attributes ^14^.

In many cases, value may be built up from information that is not directly observable but rather recalled from memory, such as sensory experiences with food items. Several findings from the fMRI literature are consistent with this perspective, demonstrating that reactivation of prior reward associations can influence future decision-making, even in independent tasks ^15– 17^. In such cases, decision-making might involve prospective simulation of outcomes that depends on the hippocampus ^18– 20^. Indeed, lesions to the medial temporal lobe and hippocampus were recently shown to lead to slower, more stochastic choices between foods, consistent with a noisier decision process ^21^. Notably, we found that VMF damage did not affect choice stochasticity or the reliability of reported preferences in a similar choice task using artwork ^22^. One possible explanation of the lack of effect of VMF damage in this task, compared with food choices, is that the value of art is assessed on directly observable (that is, visual) information and does not require prospection or construction of values from information in memory.

The effects of VMF damage on preference-based choice may depend less on the importance of this region for forming values *per se* than on its role in organizing preferences on the basis of recalled outcomes from past choices. Although VMF damage has been found to consistently increase transitivity errors during preference-based choice in several studies (that is, failing to choose A over C when A is preferred over B and B over C) ^23– 25^, this region is not necessary for the formation of coherent, transitive preferences (Yu *et al*. ^26^, 2018, preprint). Damage to the hippocampus or VMF was recently shown to affect transitive inference of paired associates in humans, implying a shared role in inferring relations between options on the basis of episodic memory more generally ^27^. Thus, the hippocampus and vmPFC may work together to stabilize decision-making based on an inferred transitive ordering of options.

## Building values toward a goal

Above, we focused on how value judgments may be constructed in relatively unconstrained settings from mnemonic, conceptual, and perceptual evidence. In this section, we discuss how contextual demands act to shape this process. More often than not, values must be adaptively tailored in response to current goals. For example, the values of potential options in a store might change if you were choosing something for yourself or for your grandmother. Identifying relevant attributes on the basis of such goals and selectively using these during value construction reduce the complexity of the decision space to make such choices tractable. Recent imaging work has uncovered putative mechanisms for selecting attributes on the basis of task demands and examined how neural value signals adapt in response to changing contexts.

Value signals in vmPFC are sensitive to the weighting of stimulus attributes on the basis of current goals. The attributes that correlate with vmPFC activity change depending on external contextual demands, such as instruction to focus on taste or health attributes of foods ^28, 29^ or to choose options with either positive or negative subjective value ^30^. The flexibility of this signal in response to arbitrary task instructions argues that this region can be tuned to the values of options as they relate to higher-order goals rather than their hedonic value independent of task.

Functional connectivity studies have shed light on how value representations may be altered by shifting demands. This work has shown that interactions between vmPFC and several other regions depend on demands for integrating information from different stimuli during valuation and decision-making. Lim *et al*. ^31^ (2013) showed that connectivity between vmPFC and the fusiform and posterior superior temporal gyri reflected the contributions of perceptual and semantic stimulus attributes to value judgments, respectively. Changes in task goals during value judgment appear to engage lateral PFC and increase connectivity between lateral PFC and vmPFC ^29, 32^. Thus, this circuit seems to be involved in shifting attention to relevant attributes for value judgment, as externally defined within a task.

A 2018 study by Tusche and Hutcherson ^33^ took a slightly different approach. They tested whether external demands to focus on specific attributes of social or dietary decisions altered the decodability of these attributes from brain activity measured by using fMRI (that is, asking whether it is possible to predict attributes of the current stimulus from the pattern of activity within a region). While the decodability of these attributes varied with task demands in lateral PFC, all stimulus attributes were decodable within an overlapping area of vmPFC across conditions. This analysis suggests that vmPFC has access to a rich representation of stimulus attributes even as task demands shift, possibly enabling a more dynamic and adaptive representation of stimulus values. In contrast, lateral PFC is more narrowly involved in specifying goal-relevant attributes.

## Learning what matters

The first section of this review focused on how values are built from attributes in unconstrained settings and the second discussed how weights on these attributes are modified depending on goals. A third (related) stream of work in the realm of reinforcement learning addresses how the value-relevance of attributes is discovered through experience.

The large space of potentially value-relevant features in the natural environment poses a challenge to making appropriate inferences about the relationships between stimuli and outcomes in the real world ^34^. In many cases, relationships may be inferred from conspicuous but incidental short-term correlations that have no bearing on the underlying causal structure of these associations. Think of the problem gambler who has come to believe that a rattling sound at the base of a slot machine predicts a jackpot rather than a loose screw. Indeed, low-level features, such as brightness, volume, or contrast, can give certain attributes greater assumed relevance when inferences are drawn about such relationships. In conditioning tasks, perceptually salient cues are more easily trained and can “overshadow” other predictors ^35^. Visual saliency biases decision-making independent of expected value ^22, 36, 37^, indicating that these low-level features can exert a powerful influence on value construction.

Directing attention to stimulus features that are predictive of rewards can effectively bootstrap the learning process by narrowing the space of features to consider in constructing option values ^38, 39^. Recent work has indicated that VMF is important for guiding attention to reward-predictive features on the basis of outcome history. We found that people with VMF damage had less of a bias to attend to a feature that was incidentally paired with reward in a visual search task, compared with healthy and frontal-damaged controls ^40^. Functional imaging has also implicated vmPFC in boosting credit for reward-predictive cues, similarly implying a role in boosting the gain on learning feature–reward associations based on their predictive history ^41^.

Examining how participants’ choices track the outcome history of attributes within a given dimension can reveal what participants have inferred about which aspects of the task predict reward. Recent neuroimaging studies have used multidimensional learning tasks to identify the regions engaged in learning attribute relevance. These studies have found that regions within the frontoparietal attention network, including lateral PFC, are engaged when identifying predictive, relevant dimensions based on feedback history ^42^. In an analogous task, vmPFC was found to encode the attentionally weighted average of the reward value of option features while switches of attention between dimensions were associated with increased activity within the frontoparietal attention network ^43^. In a similar multidimensional learning task, we tested how frontal lobe damage affected learning when participants were informed (accurately) that one low-salience stimulus dimension was predictive of reward but that two other dimensions were not. VMF-damaged participants showed worse learning about the rewardingness of the relevant dimension compared with healthy controls but did not show any increased tendency to credit rewarding outcomes to the irrelevant dimensions. In contrast, left lateral PFC damage caused an increase in the misattribution of outcomes to two (more salient) stimulus dimensions ^44^.

Just as faulty inferences might be drawn about the associations of outcomes with visual stimulus features, so too can outcomes be attributed to events that were temporally proximate but not causally related ^45^. This “spread-of-effect” phenomenon was shown to be exaggerated in non-human primates with OFC lesions ^46^, evident in a blurring of the influence of recent choices and outcomes. More recently, convergent evidence from studies of human and non-human primates with frontal lesions has indicated that damage to anterior ventrolateral PFC or lateral OFC particularly increases this failure to assign credit to the appropriate choices ^47, 48^. Functional imaging in humans similarly has pointed to lateral OFC or ventrolateral PFC as being particularly important for credit assignment when choice–outcome associations must be maintained over a delay or intervening trials ^49^. These data suggest that lateral PFC or OFC may play a similar role in specifying the features relevant to predicting outcomes in the temporal domain and the visual domain.

## Conclusions

Recent human lesion and functional imaging work has identified frontal lobe regions and related circuits that are involved in dynamically constructing the values of complex stimuli in preference-based choice, whether based on current goals or in the course of learning. The lesion results help to constrain interpretation of fMRI in healthy people, arguing for a more nuanced and specific role of VMF and lateral PFC in these processes. VMF damage leads to under-weighting specific higher-order stimulus attributes during value judgment and disrupts attention to relevant features that are predictive of rewards in the long-term during learning. In contrast, lateral PFC is involved in arbitrating between stimulus dimensions, in particular when task demands and contextual factors specify a subset of attributes relevant to a choice. Table 1 summarizes the main findings supporting this view.

**Table 1.** Overview of findings supporting the main conclusions of the article.

Frontal lobe regions	Judgment and decision-making	Reinforcement learning
Ventromedial frontal lobe (ventromedial prefrontal cortex and orbitofrontal cortex)	• Representing value-relevant attributes ^4, 6, 33^ • Forming values from attribute conjunctions ^12, 14^ • Representation of goal-congruent values ^28– 30^	• Relevance weighted value representation ^43, 50^ • Directing attention to predictive attributes ^40, 41, 44^
Lateral frontal lobe (ventral and dorsal lateral prefrontal cortex)	• Selection of goal-relevant attributes ^28, 29, 32^	• Selection of predictive dimensions ^42– 44^ • Credit assignment ^47, 48^

These findings open up new questions. For example, what is the role of VMF in incorporating complex attributes during decision-making and value judgment? One possibility is that this region is necessary for forming values out of non-linear conjunctions between attributes (that is, for interpreting the interactions between individual features that are more than the sum of their parts). Another is that VMF damage disrupts the ability to construct a latent, holistic representation of conceptual information about stimuli, leading these patients to rely instead on attribute-value representations at the level of discrete elements. The latter explanation would be broadly in line with ideas that OFC is representing information about latent task states to inform decision-making and learning ^51, 52^.

What is the nature of the interaction between lateral PFC and vmPFC during value construction? Communication between these regions appears to depend on decisions to switch or maintain attention on particular stimulus dimensions ^32, 43^. Whereas some data argue that lateral PFC acts on vmPFC to impose contextual demands on the attributes used in making value judgments ^29^, other findings support a role for vmPFC in representing “schematic” information about a task that is passed to lateral PFC to define the template for cognitive control ^53^. The direction of information flow between regions may depend on the observability of contextual cues, where directly observed contextual information may engage lateral PFC ^54^ whereas hidden changes in task state that must be inferred from schematic knowledge may rely on vmPFC or OFC ^55^.

We have focused here on the neural implementation of value construction and the particular contributions of regions of the frontal lobes to solving this problem to allow choices between the complex objects that we face in our everyday choices. However, a full account of how the brain builds holistic value representations will require a better understanding of the dynamics between frontal lobe regions and the brain areas that carry the perceptual, conceptual, and mnemonic evidence that are the building blocks of this process. Such work will have broad importance in better understanding economic and social decision behaviors and addressing how those decision processes may go awry in neurological and psychiatric illnesses.
